# Perceived Stress among Iranians during COVID-19 Pandemic; Stressors and Coping Mechanisms: A Mixed-methods Approach

**DOI:** 10.1192/j.eurpsy.2022.1274

**Published:** 2022-09-01

**Authors:** A.H. Nadoushan, M. Faghankhani

**Affiliations:** 1 Iran University of Medical Sciences, Psychaitry, Tehran, Iran; 2 Iran University of Medical Sciences, Mental Health Research Center, Tehran, Iran

**Keywords:** COVID-19, Perceived stress, Iranians, Mixed method study

## Abstract

**Introduction:**

This study was a mixed-methods study. We distributed a web-based 1scale (PSS-10), to measure perceived stress scores, through social networks from March 12 to 23, 2020. Then, we interviewed 42 students, 31 homemakers, 27 healthcare providers, and 21 male participants to identify the sources of stress and coping mechanisms.

**Objectives:**

We examined the correlates of stress among a large sample of Iranian citizens, the second country hit hard by the pandemic, and still a hot spot.

**Methods:**

This anonymous survey had 19 items falling into two sections: sociodemographic data and Cohen’s 10-item perceived stress scale (PSS-10).

**Results:**

A statistically significant difference was observed between the levels of perceived stress in individuals with different health statuses with a higher median of total PSS-10 scores reported for hospitalized individuals. The total PSS-10 scores were higher in those who were practicing self-isolation, had a relative affected with COVID-19 disease, and had experienced the death of a relative due to COVID-19 disease.

**Conclusions:**

This study highlighted the most vulnerable groups overloaded with stress in society and the sources of their stress. Furthermore, we identified the groups that perceived lower levels of stress along with their coping mechanisms. The most frequent source of stress among the most stressful groups including homemakers, students, and health care workers has directly related to their job and their principal role in this period. Abstract thought about the COVID-19 pandemic and its complications were more prevalent among students while homemakers and health care providers showed concrete thinking about the COVID-19 pandemic.

**Disclosure:**

I have no significant financial interest, consultancy, or other relationship with products, the manufacturer(s) of products, or providers of services related to this abstract?

